# Reorganisation of Brain Hubs across Altered States of Consciousness

**DOI:** 10.1038/s41598-020-60258-1

**Published:** 2020-02-25

**Authors:** D. Vatansever, M. Schröter, R. M. Adapa, E. T. Bullmore, D. K. Menon, E. A. Stamatakis

**Affiliations:** 1 Institute of Science and Technology for Brain-Inspired Intelligence, Fudan University, 200433 Shanghai, PR China; 2 Division of Anaesthesia and Department of Clinical Neurosciences, School of Clinical Medicine, UK & Wolfson Brain Imaging Centre, University of Cambridge, CB2 0QQ Cambridge, UK; 3 Department of Psychiatry, School of Clinical Medicine, University of Cambridge, CB2 0QQ Cambridge, UK; 4 Department of Biosystems Science and Engineering, Bio Engineering Laboratory, ETH Zurich, 4058 Basel, Switzerland; 5 Cambridgeshire and Peterborough NHS Foundation Trust, Cambridge Road, Fulbourn CB21 5HH Cambridge, UK

**Keywords:** Consciousness, Network models

## Abstract

Patterns of functional interactions across distributed brain regions are suggested to provide a scaffold for the conscious processing of information, with marked topological alterations observed in loss of consciousness. However, establishing a firm link between macro-scale brain network organisation and conscious cognition requires direct investigations into neuropsychologically-relevant architectural modifications across systematic reductions in consciousness. Here we assessed both global and regional disturbances to brain graphs in a group of healthy participants across baseline resting state fMRI as well as two distinct levels of propofol-induced sedation. We found a persistent modular architecture, yet significant reorganisation of brain hubs that formed parts of a wider rich-club collective. Furthermore, the reduction in the strength of rich-club connectivity was significantly associated with the participants’ performance in a semantic judgment task, indicating the importance of this higher-order topological feature for conscious cognition. These results highlight a remarkable interplay between global and regional properties of brain functional interactions in supporting conscious cognition that is relevant to our understanding of clinical disorders of consciousness.

## Introduction

Mounting reports now indicate that spontaneous brain activity patterns at rest are organised into large-scale networks with unique profiles of functional interactions between distinct brain regions^1,2^. Extending across both unimodal and transmodal cortices^3,4^, these spatiotemporal correlation patterns are suggested to represent an intrinsic organisational feature of brain-wide communication that is necessary for healthy and adaptive cognitive processing^5^ with conscious awareness^6,7^ (hereinafter referred to as “conscious cognition”). In fact, marked changes in brain functional connectivity architecture have been observed during altered states of consciousness in non-rapid eye movement (NREM) sleep^8,9^, pharmacologically-induced sedation^10–12^, and neurological disorders that result in pathological shifts in consciousness^13^. Taken together, this converging body of evidence indicates a central role played by ongoing brain connectivity patterns in maintaining conscious cognition, which requires further empirical investigation.

To this end, the application of graph theoretical methods to resting state functional magnetic resonance imaging (fMRI) data has provided an alternative approach to statistically describe the topological features of brain functional networks and their relation to conscious cognition^14^. Considering cortical regions as nodes and functional interactions amongst them as edges, previous studies have shown that brain graphs possess a non-random modular organisation, balancing a level of multi-modal integration and segregation between distinct functional subunits^15^ that not only depicts transient modifications during cognitive task performance^16^, but also predicts individual responses to cognitive training^17^. Furthermore, carrying fundamental importance within these global architectural features, brain graphs were also shown to harbour cortical “hubs”^18,19^ – regions with high strength or centrality, which have a tendency to connect to each other, forming a so-called “rich-club” organisation for efficient information transfer^20^. Traversing across both transmodal (e.g. default mode and dorsal attention) and unimodal (e.g. visual, auditory and sensorimotor) cortices^20^, emerging reports now highlight the relevance of brain hubs and rich-club collectives for a diverse set of higher-cognitive processes^21,22^, and for the emergence of a conscious mind across development^23^.

In agreement with this notion, a recent study comparing patients in coma to healthy participants revealed a marked reorganisation of both transmodal and unimodal brain hubs^24^. Similarly, progressively greater disturbances to the connectivity strength of both primary somatosensory and association cortices were observed in hepatic encephalopathy patients with systematic differences in their levels of consciousness, that ultimately resulted in gross reorganisation of brain network topology^25^. Further adding to these findings, loss of consciousness with pharmacological interventions in healthy participants has been linked to reduced functional integration in the brain, affecting both the frontal and parietal cortices^26^. Such changes were attributed to an increase in local clustering and small-worldness^10^, specifically affecting brain hubs that potentially tips the balance in favour of more local processing as opposed to globally coupled brain activity dynamics^27^.

Though limited, the evidence provided by these studies underlines that prominent architectural features of brain graphs, such as brain hubs and rich-club organisation, may serve as anatomical and functional substrates that provide a scaffold for conscious cognition. Specifically, they might represent the neural embodiment of the theorized “global workspace” for the efficient broadcasting and circulation of information across the brain, which is suggested to be an essential prerequisite of a conscious mind^7,28,29^. However, beyond the gross disturbances observed with loss of consciousness in prior studies, changes in both global and regional architectural features of brain graphs during systematically reduced levels of consciousness in healthy participants requires further examination in order to ascertain a direct link between complex brain network topology and conscious cognition.

Here we assess modifications to the whole-brain functional interactions in response to the pharmacological agent propofol using resting state fMRI. Specifically, we set out to investigate alterations in the community structure (modularity)^15^ of fMRI-based brain graphs and the reorganisation of cortical hubs and rich-clubs^19,20^ across baseline rest, as well as light and moderate levels of propofol-induced sedation. Propofol is an anaesthetic agent that acts on GABA_A_ receptors to increase neuronal inhibition^30^. In small doses, propofol induces hypnotic sedation similar to that observed in deep sleep^31^, acting on key brain regions commonly implicated in our ability to monitor and make sense of the world around us^32^. In this study, distinct levels of propofol-induced sedation were associated with an overall stability in the global features of brain functional network architecture, however, with a marked reorganisation of brain hubs across parametric reductions in conscious cognition. Furthermore, such radical reshuffling coincided with decreases in the strength of rich-club collectives that was significantly associated with behavioural performance in a semantic judgement task. Such demonstration of a link between changes in brain functional connectivity patterns and cognitive task performance could provide additional supportive evidence for the proposed functions ascribed to highly-connected brain regions for normal cognitive processing and inform the framework for understanding their dysfunction in disorders of consciousness.

## Results

### Preservation of modular architecture across propofol-induced sedation

Based on previous evidence suggesting alterations in global information processing with sedation-induced loss of consciousness^33^, our initial analysis investigated potential changes in the modular organisation of whole-brain functional interactions with systematic increases in propofol-induced sedation. For that purpose, we calculated the Louvain modularity index (*Q*) across a range of cost densities on individual brain graphs and determined the corresponding network partitioning.

**Figure 1:**
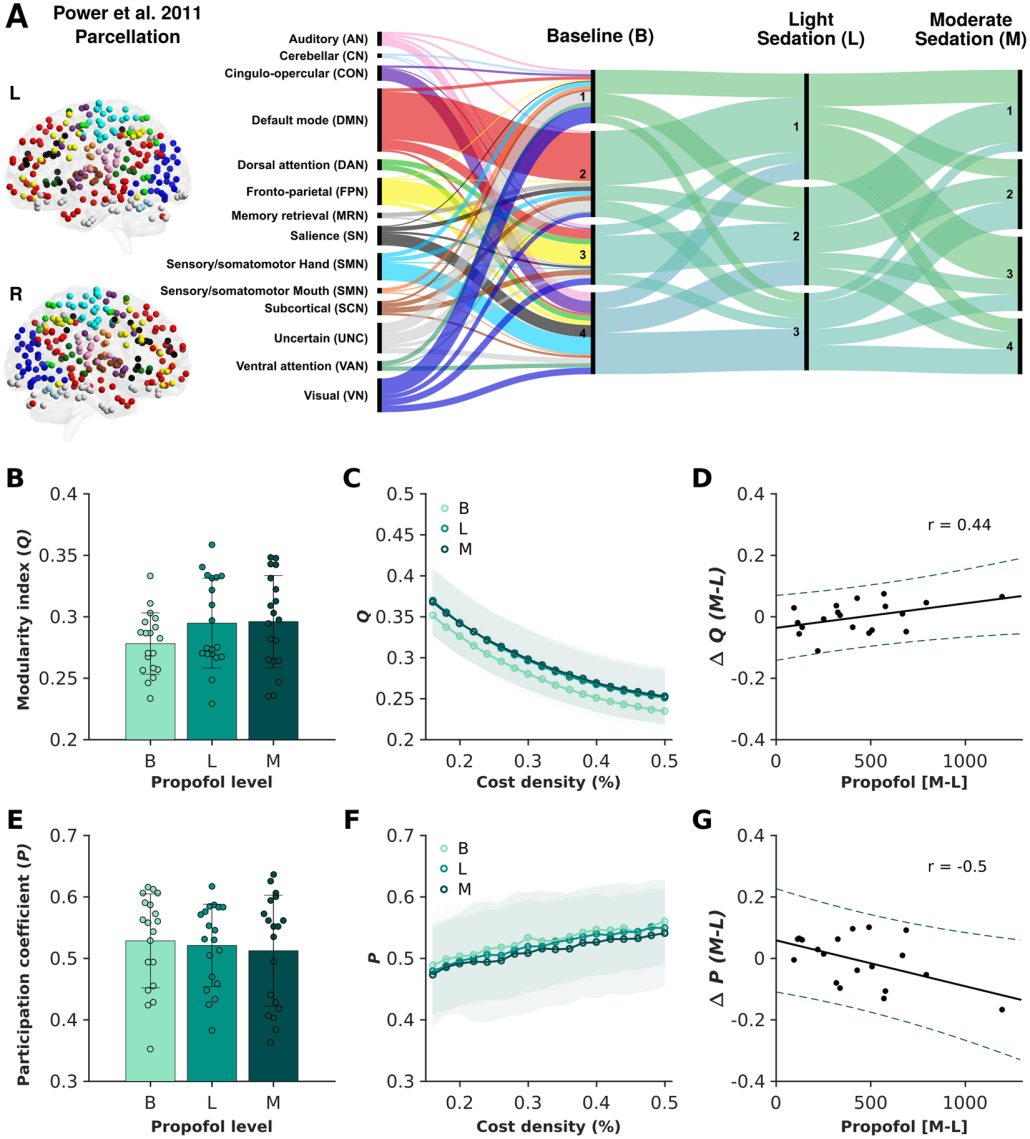
Preservation of the modular brain network organisation across baseline and two stages of propofol-induced sedation. Based on the consensus partitioning of individual brain graphs across the chosen cost densities (16 to 50% in 2% increments), between three to four broad communities were identified in all three experimental conditions (panel A). The corresponding Alluvial diagram shows the flow in the consensus community affiliations of 258 regions of interest (ROIs) from a previously introduced parcellation scheme (Power *et al*.^61^) across baseline resting state, as well as light and moderate propofol-induced sedation conditions. Modularity index (*Q*) and participation coefficient (*P*) did not show statistically significant alterations across experimental conditions (panels B,E) and across the chosen range of cost densities (panels C,F). Bars represent the standard error with the distribution of individual values provided for each measurement across each experimental condition. Notably, there was a significant correlation between the change in modularity index (*Q*) (r = 0.44, p = 0.029; panel D) as well as the change in participation coefficient (*P*) (r = −0.50, p = 0.014; panel G) and blood plasma propofol concentration when comparing the two propofol-induced sedation conditions at the percolation threshold (16% cost density). While straight lines indicate the linear fit, dotted lines represent the 95% confidence intervals.

At baseline resting state, the consensus partitioning of whole-brain functional interactions revealed four distinct communities dominated by both transmodal (e.g. default mode and fronto-parietal networks), as well as unimodal regions (e.g. visual, and sensory/somatomotor networks) (Fig. 1A). Across the three experimental conditions, despite minor changes evidently present in the community affiliation of brain nodes, a consistent global network topology with three to four distinct communities was identified. In line with this observation, at the individual level, there were no significant differences in the modularity index (*Q*) (F_(1.96,35.28)_ = 1.87, p = 0.17) or average participation coefficient (*P*) (F_(1.51,27.10)_ = 0.28, p = 0.70) across baseline, light and moderate sedation conditions (Fig. 1B,C,E,F), potentially reflecting the preserved (albeit reduced) levels of conscious awareness in this study.

However, a significant positive correlation was observed between the change in blood plasma propofol concentration and the change in modularity index (*Q*) when comparing the moderate and light sedation conditions at the percolation threshold (r = 0.44, p = 0.029) (Fig. 1D). Further interrogating this link within the two distinct sedation conditions, we found no relationships under light sedation (r = 0.10, p = 0.33), but a significant positive correlation between propofol concentration and modularity index (*Q*) under moderate sedation (r = 0.54, p = 0.0092). In parallel, the change in blood plasma propofol concentration between moderate and light sedation conditions was negatively related to the change in participation coefficient (*P*) (r = −0.50, p = 0.014) (Fig. 1G). While no correlation was observed under light sedation (r = −0.19, p = 0.22), a significant negative correlation was present between propofol concentration and participation coefficient (*P*) under moderate sedation (r = −0.68, p = 0.00071).

Overall, the results indicate the preservation of global brain functional network interactions, with minor differences observed in the community partitioning and diversity of nodal affiliations across systematic alterations in the levels of consciousness. Specifically, in the moderate sedation condition, individual differences in the blood plasma concentration of propofol was predictive of the participants’ brain functional network topologies, indicating differential alterations in the global brain network architecture with distinct levels of propofol administration.

### Reorganisation of brain hubs across propofol-induced sedation

Given prior reports that demonstrated differences in the hubness of network nodes with loss of consciousness^10,24,33^, we next calculated the hub disruption index across three experimental conditions with the aim of investigating the potential reorganisation of brain hubs in response to systematic reductions in consciousness. Hub disruption index is a summary metric that characterises regional changes in the hubness of network nodes in response to external manipulations^24,25^.

**Figure 2:**
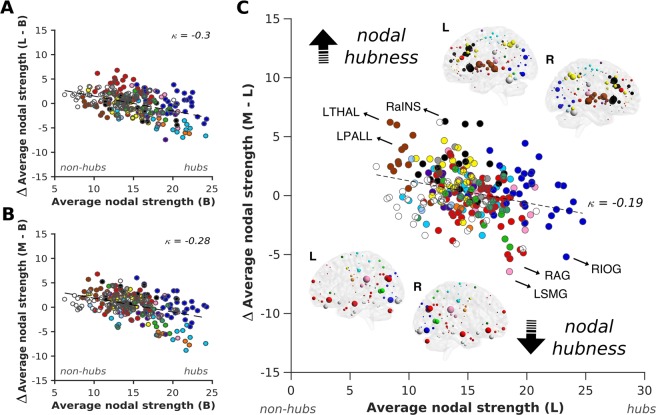
Reorganisation of brain hubs with propofol-induced sedation. A radical reorganisation of brain hubs was observed across the two levels of propofol-induced sedation as compared to the baseline resting state condition. *κ* was calculated as the slope of a linear fit to the scatterplot of average functional connectivity strength between a chosen condition (light or moderate) and the difference between this condition and baseline resting state recording for each participant (panels A,B). When directly assessing sedation-level-dependent disruptions to the hub structure between moderate and light sedation conditions, regions belonging to the transmodal default mode (e.g. RAG = right angular gyrus), as well as the unimodal visual (e.g. RIOG = right inferior occipital gyrus), auditory (e.g. LSMG = left supramarginal gyrus) and sensory/somatomotor networks showed a marked decrease in their hubness, while those assigned to the subcortical (LTHAL = left thalamus, LPALL = left pallidum) and salience (RaINS = right anterior insula) networks displayed a strong increase in their nodal strength (panel C). Network nodes are colour-coded based on the Power *et al*.^61^ parcellation scheme. The size of nodes on the MNI glass brains indicates the magnitude of change in the nodal strength for each brain region. While the top glass brains show increase in nodal hubness, the lower glass brains indicate decreases in nodal hubness with the same external manipulation.

Across participants, the group average hub disruption index (*κ*) showed a negative slope between all three comparisons (B-L, B-M, and L-M), illustrating the marked reorganisation of network hubs with systematic increases in propofol administration and reduced levels of consciousness (Fig. 2). These results suggest that the brain hubs showed the greatest decrease in their strength with more propofol administration, whereas non-hub nodes displayed a tendency to increase their functional interactions. Specifically, when comparing the light and moderate sedation conditions against the baseline resting state recording, such alterations were most evident for several regions of the visual, auditory and sensory/ somatomotor networks, which all decreased their hubness. On the other hand, subcortical network regions increased their hubness with the same external manipulation (Fig. 2A,B).

More importantly, when directly comparing sedation-level dependent disruptions to the hub structure between the moderate and light sedation conditions, a decrease in hubness was observed in a number of regions assigned to the transmodal default mode network (e.g. right angular gyrus), as well as the unimodal visual (e.g. right inferior occipital gyrus), auditory (e.g. left supramarginal gyrus), and sensory/somatomotor networks, whereas, regions belonging to the subcortical (e.g. left thalamus and pallidum) and salience (e.g. right anterior insula) networks increased their hubness (Fig. 2C). Overall, the results indicate a radical reorganisation of brain hubs with systematic alterations in propofol-induced sedation.

### Reductions in rich-club strength relate to cognitive performance

In addition to the observed changes in the hubness of network nodes across three experimental conditions, we next set out to investigate potential alterations in the rich-club organisation, namely, the changes in functional interactions that link individual brain hubs. The rich-club coefficient denotes the tendency of highly connected hub regions to connect more preferentially to themselves as opposed to regions with low strength, thus alluding to an important higher-level topology of the brain^20^ that might constitute a supportive backbone for communication across distinct brain regions.

**Figure 3:**
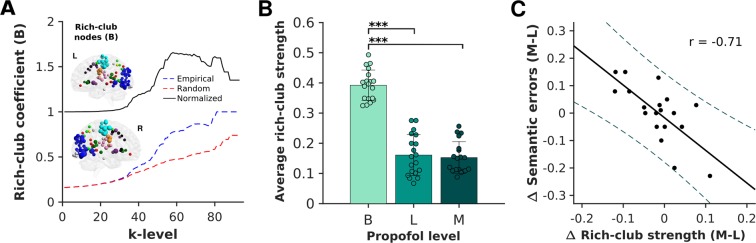
Reductions in rich-club connectivity strength across propofol-induced sedation relate to semantic processing. Calculated on the average brain graph of the baseline resting state condition, a rich-club organisation was observed with an increasing rich-club coefficient >1 that was significantly different from surrogate networks (*n* = 100) between *k*-levels of 7–58. At the individual level, this rich-club organisation encompassed both transmodal cortices such as those that belong to the default mode, dorsal-attention and cingulo-opercular networks, as well as unimodal regions that belong to the visual, auditory and sensory/somatomotor networks. The size of nodes on the MNI glass brain indicates the number of participants in which the node was identified as part of the rich-club organisation (panel A). The strength of rich-clubs across participants showed a significant decrease between baseline and light (p < 0.001) as well as baseline and moderate sedation conditions (p < 0.001), with no statistical differences observed between the light and moderate sedation conditions (p = 0.99) (corrected for multiple comparisons using the Bonferroni method). The bars represent the standard error with the distribution of individual values provided for each measurement across each experimental condition (panel B). A Pearson correlation indicated that the change in rich-club strength was significantly related to the change in the participants’ errors in the semantic judgment task between moderate and light sedation conditions (r = −0.71, p = 0.00042). While straight lines indicate the linear fit, dotted lines represent the 95% confidence intervals (panel C).

Prior to tracking rich-club strength across experimental conditions (B, L, M) for each individual, we first examined the existence of a rich-club architecture on the average brain graph in the baseline resting state condition. For that, we binarized the average brain graph at the percolation threshold (16% cost density; baseline condition) and calculated rich-club coefficients across a set of *k*-levels, ranging from *k* = 1 until the maximal possible degree (*k*_max_). During the baseline resting state condition, a significant rich-club organisation was observed between *k*-levels ranging from *k* = 7 to 58, with a normalised rich-club coefficient >1 (Fig. 3A). The same analytical procedure was then carried out for each subject. All *k*-levels above the upper *k* bound with a significantly different rich-club coefficient as compared to the surrogate networks were denoted as rich-clubs. In line with previous investigations^18,20^, the common rich-club nodes across all participants spanned both transmodal cortices such as those that belong to the default mode, dorsal-attention and cingulo-opercular networks, as well as unimodal regions that belong to the visual, auditory and sensory/somatomotor networks (Fig. 3A).

Analysis at the individual level demonstrated a significant reduction in the rich-club strength across the three experimental conditions (F_(1.70, 30.65)_ = 135.87, p < 0.0001) (Fig. 3B). Post-hoc t-tests indicated a significant difference between the baseline and light (p < 0.0001), as well as baseline and moderate sedation conditions (p < 0.0001), while no significant difference was observed between light and moderate sedation (p = 0.99) (Fig. 3C). More importantly, changes in the functional connectivity strength of participants’ rich-club organisations between the two distinct propofol-induced sedation conditions were significantly associated with changes in their behavioural performance in a semantic judgment task, in which they were asked to categorise presented words into living or non-living items. Participants who showed a greater reduction in their rich-club connectivity strength, displayed a greater increase in the errors that they committed during task performance (r = −0.71, p = 0.0007) (Fig. 3D). Together with the lack of significant correlations observed between the change in blood-plasma propofol concentration and rich-club strength (r = 0.17, p = 0.25) as well as semantic errors (r = 0.16, p = 0.26), the observed findings suggest that this higher-order topological feature may reflect the effect of pharmacological intervention on cognitive processing as opposed to that of propofol levels.

## Discussion

The overall objectives of this study were to interrogate modifications to the brain functional network topology of healthy participants during systematic alterations of levels of consciousness, and to assess the relevance of complex brain network organisation to conscious cognition. The focus was hereby on the global modularity, regional hub structure and rich-club organisation of brain graphs in response to parametric increases in propofol – an anaesthetic agent that is suggested to act on the inhibitory GABA_A_ receptors^32^ and is often used clinically to reduce levels of consciousness in patients^30^.

Across the three experimental conditions studied here, brain graphs displayed a relatively persistent modular architecture with insignificant alterations in the community partitioning and diversity of nodal affiliations. However, in contrast to the observed preservation of global network topology across propofol-induced sedation, there was a substantial reorganisation of regional brain hubs that resulted in decreased connectivity strength within both transmodal (e.g. default mode) as well as unimodal (e.g. visual, auditory, sensory/somatomotor) brain regions, and an increase in the strength of connections made by subcortical and salience networks. In parallel, a significant reduction in the connectivity strength of rich-club nodes was observed that was predictive of the individual variability in behavioural performance during a semantic judgment task. Overall, the results of this study suggest the resilience of global topological features with a marked reorganisation of regional brain hub connections as well as rich-club organisation at light and moderate levels of sedation.

Applying pharmacological agents to induce altered levels of consciousness, previous studies have largely focused on changes in whole-brain functional interactions between normal wakefulness and anaesthetic dosages that induced loss of consciousness. For example, Schrouff and colleagues have previously reported on a global decrease in whole-brain functional integration within and between distinct large-scale brain networks with loss of consciousness^26^. In parallel, our group has demonstrated an increase in normalized local clustering and a profound decline in long-range thalamo-cortical as well as cortico-cortical connectivity during loss of consciousness as compared to wakeful rest^10,34^. Monti and colleagues on the other hand, have suggested a level of stability in the graph metrics between baseline and sedation conditions, but with a marked decline in the characteristic path length during loss of consciousness that was uniquely associated with a decrease in global information processing efficiency^33^. Taken together, these results may be indicative of less cross-modular communication between brain systems, i.e. more segregated processing of information with pharmacologically-induced loss of consciousness.

It is important to note that, as compared to the baseline resting state condition, our results have also indicated a trend towards greater modularity (Q) and reduced participation coefficient (P) in the two levels of propofol-induced sedation as well as a link between these measures and the change in blood plasma propofol concentration. Mirroring the results of previous investigations, these findings suggest more segregated processing and less diverse interactions between communities in altered levels of consciousness across participants^10,26,27^. However, the lack of a significant condition-specific difference in these metrics and the overall stability of global functional interactions observed in our study may reflect the relative persistence of consciousness albeit at different stages of propofol-induced sedation^33^. Further studies with comparable sedative administration, graph construction and analysis techniques will be required to make conclusive inferences on the differential influence of loss versus reduction of consciousness on the whole-brain connectivity architecture. Nonetheless, the results of this study not only highlight the paramount importance of differentiating between these states in studies of pharmacologically induced sedation, but also emphasize the necessity for interrogating regional contributions to whole-brain functional interactions beyond that of global architectural features.

Despite the observed resilience of global brain functional network topology to propofol-induced sedation, however, the hub disruption index revealed a marked reorganisation in the connectedness of individual brain regions across the three experimental conditions. In response to a shift from baseline resting state to light and moderate levels of sedation, primary sensory/somatomotor network regions with high nodal connectivity strength showed notable reductions in their functional interactions, whereas non-hub subcortical regions demonstrated increases with the same external manipulation. Importantly, when directly comparing light and moderate sedation conditions, regions belonging to the transmodal default mode (e.g. right angular gyrus), and unimodal visual (e.g. right inferior occipital gyrus), auditory (e.g. left supramarginal gyrus), sensory/somatomotor networks reduced their hubness towards higher levels of propofol sedation, while regions belonging to the subcortical (e.g. thalamus and pallidum) and salience networks (e.g. right anterior insula) increased their hubness.

Constituting one of the most studied large-scale brain networks, the default mode network integrity has been consistently associated with levels of consciousness in prior research^35,36^. Significant reductions in the functional connectivity among core nodes of the default mode network, including the posterior cingulate and medial prefrontal cortices, have been reported throughout deep NREM sleep^37^. Similar results have also been observed in studies using pharmacological agents^36^, in which propofol^11,12,26^, sevoflurane^38^ and midazolam^39^ induced sedation have all been linked to decreases in both within and between-network interactions of the default mode network, specifically to the primary sensory/somatomotor brain regions. Although earlier studies have revealed no consistent alterations in the topography of other large-scale brain networks with pharmacological interventions^36^, emerging studies now indicate the ability of the auditory and visual network hubs’ functional interactions to differentiate between minimally conscious and vegetative state patients^40,41^, and underline the default mode-motor functional interactions as a loci of disturbance in propofol-induced sedation^12^. Furthermore, given previous evidence on the disintegration of salience network connections with loss of consciousness^42^, its proposed role in the modulation of interactions between default mode and fronto-parietal control networks^43^, as well as recent investigations indicating the mediatory effect of sparsely connected brain regions on brain connectivity alterations^44^, the observed increase in these regions’ hubness during propofol-induced sedation in our study might reflect their importance in maintaining conscious awareness at play that needs to be further investigated.

Initially described as a network of regions that deactivate during goal-oriented tasks^45^, more recent studies now illustrate an association between the default mode network and memory-based cognitive paradigms including working^16^, semantic^46^ and episodic memory^47^ that collectively form crucial components of a conscious mind. With its extensive structural and functional connections to the rest of the brain^18,48^ and its strategic hierarchical positioning along a principal gradient of macro-scale cortical organisation^4^, the default mode network in this context is believed to play a “global integrator” role for the multi-modal integration of information^16,49,50^, potentially constituting a part of the theorised “global workspace” for efficient transfer of information amongst distinct functional subunits. Hence, taken together with marked reductions observed in the functional interactions of both the transmodal (e.g. default mode) and unimodal (e.g. visual) regions in our study, such reorganisation of brain hubs might indicate a dysfunction related to this integrative machinery. Thus, further research into the modification of brain hub structure in complex brain graphs with physiological, pharmacological and pathological alterations will be required to obtain a more complete picture of the neural correlates of consciousness.

Further highlighting this point, brain hubs have been suggested to support communication amongst not only within, but also across functionally specialised brain regions^21,51^. A higher-order topology that characterises this function is provided by the so-called “rich-club organisation” that is suggested to enable efficient information transfer^20^. To this end, we first confirmed the existence of a rich-club in the baseline resting state condition that comprised both transmodal default mode, cingulo-opercular, dorsal/ventral attention networks, as well as unimodal visual, auditory, and sensory/somatomotor networks. Importantly, there were significant decreases observed in the strength of rich-club connectivity following propofol-administration as compared to baseline resting state scanning, potentially highlighting an important decrease in the level of efficiency for multi-modal integration of information to support conscious cognition.

In line with this interpretation, the change in the strength of rich-club connectivity between light and moderate sedation conditions was correlated with errors committed in the semantic judgment task, potentially indicating a disturbance to the level of transmodal integration required for the conscious processing of semantic information^52^. Similarly, the efficiency of information transfer provided by this complex organisation of brain hubs was previously associated with the degree of general intelligence^20,51^ with a recent meta-analysis indicating a role for rich-club regions in enabling a diverse set of cognitive processes^21^. In addition, more recent evidence on the strengthening and modification of the brains’ functional rich-club organisation from childhood to adulthood^22,23^ further advocates for the central importance of higher-level architectural organisation of the brain in the conscious processing of information, which will require further investigation.

In conclusion, our study highlights that propofol-induced sedation is associated with a global stability of whole-brain functional interactions, yet a marked reorganisation of the hubness and rich-club organisation that may be linked to the conscious processing of information. Nevertheless, future studies that examine such topological changes during the performance of cognitive tasks under reduced levels of consciousness will be required to further delineate the exact contribution of brain functional interactions to supporting and maintaining consciousness. As well as improving our understanding of the neural correlates of conscious information processing, the outcome of this study further implicates the whole-brain network topological features as crucial components of a brain architecture that can support a healthy and adaptive mind.

## Methods and Materials

### Participants and study protocol

The study was approved by the Cambridgeshire 2 Regional Ethics Committee and informed consent for participation was obtained from 25 right-handed healthy individuals, in accordance with relevant guidelines and regulations outlined in the Declaration of Helsinki. With the aim of investigating changes in brain functional connectivity architecture with parametrically altered levels of consciousness, eyes-closed resting state fMRI data and behavioural responses to a subsequently administered semantic judgment task were obtained under three distinct experimental conditions: (i) at baseline, (ii) at light propofol-induced sedation and (iii) at moderate propofol-induced sedation. A total of six subjects were excluded prior to the functional connectivity analysis, one due to the lack of whole-brain coverage during image acquisition, and five due to excessive motion as identified by our comprehensive data denoising procedures described below. The mean age for the final group of 19 participants included in this study was 35.16 (SD = 8.88), ranging from 23 to 52 with 12/7 female to male ratio.

Participants were informed about the risks and effects associated with propofol administration, intravenous cannulation, blood sampling and MRI scanning. Using a computer controlled intravenous infusion, we aimed to achieve three general stages of target plasma levels of propofol including no drug (*baseline, B*), 0.6 µg/mL (*light sedation, L*), 1.2 µg/mL (*moderate sedation, M*). With the aim of reaching plasma and effect-site propofol concentration equilibrium, a period of 10 minutes was allowed before the resting state fMRI runs commenced. Two blood samples (2 × 1 mL) were collected at each sedation stage for later measurement of plasma propofol concentrations with high performance liquid chromatography (HPLC) that confirmed the correct categorisation of the functional runs into two general stages of propofol-induced sedation. Furthermore, levels of conscious awareness were examined verbally immediately before and after each scanning run. A detailed explanation of the propofol administration and a justification behind the use of fixed target propofol concentrations have been outlined elsewhere^53^.

### MRI data acquisition

The experiment was conducted in a Siemens TIM Trio 3T scanner at the Wolfson Brain Imaging Centre (WBIC), Cambridge. The imaging protocol was initiated with a T1-weighted structural scan (MPRAGE sequence) using 1 mm isotropic resolution in the sagittal plane (TR = 2250 ms; TI = 900 ms; TE = 2.99 ms; flip angle = 9°; field-of-view (FOV) read = 256 mm; slices per slab = 176). For the fMRI runs at each of the three experimental conditions (i.e. baseline, light sedation, and moderate sedation), the functional volumes were acquired using an echo-planar imaging (EPI) sequence that consisted of 32 interleaved, descending, oblique axial slices, 3 mm thick with an interslice gap of 0.75 mm and an in-plane resolution of 3 mm (FOV-read = 192 mm, TR = 2000 ms, TE = 30 ms, flip angle 78^o^, with 145 volumes i.e. approximately 5 minutes). Parts of this dataset have been previously employed in our prior publications^12,44^.

### MRI data preprocessing

The pre-processing and image analysis were both performed using Statistical Parametric Mapping (SPM) Version 12.0 (http://www.fil.ion.ucl.ac.uk/spm/) and MATLAB Version 17a (http://www. mathworks.co.uk/products/matlab/). The first five volumes were removed to eliminate saturation effects and achieve steady state magnetization. The remaining data were slice-time adjusted, motion corrected, normalized to the Montreal Neurological Institute (MNI) space by utilising the co-registered and segmented high-resolution grey matter structural image and *a priori* templates^54^. No spatial smoothing was employed given recent evidence suggesting significant influence of this process on the subsequent estimation of graph theoretical metrics^55^.

The *Conn* functional connectivity toolbox^56^ was used for the fMRI data denoising procedures and the construction of brain graphs. First, *CompCor*, a strict noise reduction method, was utilised to remove data components attributable to the signal from white matter and cerebrospinal fluid^57^. This eliminated the need for global signal normalisation, which has been reported to introduce spurious anti-correlations^58^. The subject-specific six rigid-body realignment parameters and their second order derivatives were also included in the analysis as potential confounds^59^. With the aim of further removing motion artifacts, a scrubbing procedure was employed, which identified and censored volumes with excessive motion^60^. For that purpose, a composite motion score was calculated based on normative thresholds (i.e. global signal change greater than z = 9 and head motion greater than 2 mm). As per our exclusion criteria, five participants with more than 15% of invalid volumes in any of their three functional runs were removed from further functional connectivity and graph theoretical analyses. Moreover, a temporal filter of 0.008 and 0.09 Hz was applied to focus on low-frequency fluctuations^2^. The resulting images were then used to construct brain graphs for each experimental condition.

### Brain graph construction

The main objective of this study was to assess potential alterations in the topological organization of functional connectivity, and in particular, changes in whole-brain modular organization, hubness and rich-club architecture across baseline resting state recording, and more importantly, two levels of propofol-induced sedation. Thus, for graph construction we employed a whole-brain approach, in which correlation matrices based on pre-defined ROIs formed the basis of our graph theoretical analysis^61^.

#### Regions of interest definition

The set of 264 ROIs was adopted from the parcellation scheme made publicly available by Power *et al*.^61^. As opposed to voxel-wise or anatomical definitions, the selected set of ROIs minimises signal overlap from multiple functional regions. The network partitions outlined in this publication were utilised to assign each ROI to one of the 14 large-scale networks. This included 10 well-established networks covering dorsal (DAN) and ventral attention (VAN), salience (SAN), cingulo-opercular (CON), fronto-parietal control (FPN), default mode (DMN), visual (VN), auditory (AN), sensory/ somatomotor (hand and mouth) (SMN), subcortical networks (SCN), and two networks that fall into memory retrieval, and cerebellum, as well as a remaining set of nodes that could not be assigned to any of the above groups. Six ROIs (one from DAN and five from SMN) were removed from the analysis due to incomplete brain coverage, resulting in a total of 258 ROIs among 14 network partitions that were ultimately used in this analysis.

#### Functional connectivity

For each experimental run and for each participant, all-to-all undirected functional connectivity matrices were constructed for subsequent graph theoretical analyses. For this, we calculated Pearson correlation coefficients (r) between average blood oxygen level dependent (BOLD) signal time series obtained from spheres (6 mm in radius) placed on the MNI coordinates of all 258 ROIs. Given recent reports that suggest the potential importance of anti-correlations in the functional connectivity of healthy brain processing^62^, our main analyses included both positive and negative weights, where applicable^63^. Additionally, network properties were computed and compared across a range of 18 proportionally thresholded brain graphs, starting with the percolation threshold (i.e. the network density at which the matrices were fully connected across all runs and all participants) until 50% network density was reached in 2% increments.

### Graph theoretical analysis

All graph theoretical metrics were calculated using MATLAB functions obtained from the publicly available Brain Connectivity Toolbox (downloaded 2017-15-01)^64^. Specifically, we employed graph metrics for the modularity of brain graphs^65^, nodal participation coefficients^66^, rich-club organization^67^, and hub disruption index^24^ with the aim of investigating both global and regional topological changes in functional connectivity across baseline rest and two distinct levels of propofol-induced sedation.

#### Modularity

The application of graph metrics to brain functional connectivity networks has previously revealed topological features that are common across many biological systems^14^. One such feature that has drawn considerable attention in the network neuroscience literature is modularity, i.e. the partitioning of a brain graph into subsets of nodes, or communities, that are strongly inter-connected among themselves, but less strongly coupled to other communities. Modularity is believed to signify a core organisational feature of both anatomical and functional brain networks, denoting the relative functional specialisation or segregation of individual brain regions as well as the integration of information^15^. Here, we used a standard approach to infer the community structure of brain graphs, also known as modularity maximisation, which aims to partition a network’s nodes into non-overlapping communities so as to maximise a quality function (*Q*)^64,65^.

Utilising the Louvain modularity maximisation algorithm, the modularity index (*Q*) was calculated (γ = 1) for all individual weighted matrices across 18 different network densities (16% percolation threshold to 50% connection density in 2% intervals). Negative values were treated asymmetrically as compared to the positive connections^63,65^. Given the stochastic nature of the algorithm, the maximum modularity index (*Q*) over 10 iterations was chosen as the optimal community partitioning. Moreover, we calculated the nodal participation coefficient (*P*) across the chosen range of connection densities. The participation coefficient characterises the level at which a node of an assigned module connects with nodes of other communities, denoting the diversity of inter-modular connections and the nodes’ integrative role across communities^64,66^.

#### Hub disruption index

Following up on previous research that has indicated the selective influence of pharmacologically-induced sedation on brain regions comprising high levels of functional connectivity^10,33^, we investigated changes in the profile of whole-brain functional connectivity hubs in response to propofol-induced light and moderate sedation. For this purpose, we applied the hub disruption index (*κ*)^24,25^, which provides a systematic characterization of changes in the overall organization of brain hubs across experimental conditions. In the present study, *κ* was calculated as the slope of a linear fit to the scatterplot of group average nodal strengths (sum of Pearson correlation values) between a chosen condition, and the difference between this condition and either of the subsequent experimental conditions for each participant.

#### Rich-club organisation

A rich-club organization is defined as the tendency of high degree (*k*) nodes to connect to each other more strongly than expected by their degree alone^67^. In the brain, rich-club nodes are suggested to support the efficient distribution of information across functional modules and serve as a relay for cortical communication^20^. The rich-club coefficient (ϕ), was calculated as the fraction of the number of existing inter-areal (binary) edges for brain regions with a degree larger than (*k*), divided by the number of possible connections among these nodes. Each rich-club coefficient was normalized using 100 surrogate networks. To assess alterations in the rich-club connectivity across experimental conditions, we first interrogated the existence of a rich-club organisation on the group average matrix of the baseline resting state (the matrix was binarised at the percolation threshold). Using permutation testing, we then identified *k*-levels at which the empirical values significantly differed from the distribution of surrogate rich-club coefficients. Next, we defined the maximum *k*-level at which the empirical ϕ value was found to be significantly higher than the rich-club coefficients of surrogate networks as the “baseline rich-club”. This procedure was then carried out at the individual level and the average strength of connections between the defined baseline rich-club nodes across the three experimental conditions was calculated.

### Statistical analyses and behavioural correlation

The Louvain modularity index (*Q*) (averaged across cost density thresholds) and the participation coefficient (*P*) (averaged across nodes and cost density thresholds) across the baseline and two levels of propofol-induced sedation conditions were statistically compared with repeated measures ANOVAs and post hoc t-tests to investigate significant changes (*p* < 0.05 significance level, corrected for multiple comparisons using the Bonferroni method). In addition, we probed the relationship between individuals’ changes in blood plasma propofol concentration and changes in brain graph modularity index (*Q*) and participation coefficient (*P*) between the light and moderate propofol-induced sedation conditions using Pearson correlations.

Furthermore, the connectivity strength of rich-club nodes during baseline was compared to the nodal strength among these nodes during the light and moderate sedation conditions using repeated measures ANOVAs and post-hoc t-tests. Finally, we investigated a potential link between the changes in the strength of rich-club connectivity to the participants’ behavioural performance on a semantic decision task^53^, carried out inside the scanner following the resting state fMRI sessions within the described stages of propofol-induced sedation. In these 5.5 minutes task runs, participants were audially presented with five 30 s blocks of living and non-living words that were alternated with five 30 s blocks of acoustically matched non-words (buzz/noise), using the Cognition and Brain Sciences Unit Audio Stimulation Tool (CAST). In each block, a total of eight 3 s stimuli was presented with stimulus onset asynchrony (SOA), followed by a 6 s of silence. ﻿While words were pseudo-randomly drawn from groups of living (e.g. tiger, birch) and non-living items (e.g. table, stone) that were matched for various psycholinguistic variables, non-word buzz/noise items were generated from word stimuli matched for average spectral profiles. Participants were instructed to indicate with a button press whether the presented stimuli were living/non-living items or buzz/noise-type items. Further details about this task have been provided elsewhere^53^, in which increases in errors during living/non-living judgment were reported to be a strong indicator of conscious processing. Finally, using a Pearson correlation, we assessed the relationship between the change in the rich-club strength and the change in error rate in the semantic judgment task between the light and moderate sedation conditions.

### Visualisation

For the modularity analysis, the consensus partitioning across all participants in each experimental condition was visualised on an Alluvial diagram as implemented in RAWgraphs (http://rawgraphs.io/). The average consensus partitioning across all network densities and all participants was calculated using an association-reclustering framework over 10 iterations^68,69^. The average hub disruption index across subjects and the rich-club nodes across participants were visualised on MNI glass brains using BrainNet Viewer^70^. The remaining graphs were constructed using MATLAB visualisation functions.

## Data Availability

The datasets generated and/or analysed during the current study are available from the corresponding authors on reasonable request.

## References

[1] Biswal B, Yetkin FZ, Haughton VM, Hyde JS (1995). Functional connectivity in the motor cortex of resting human brain using echo-planar MRI. Magnetic Reson. Med..

[2] Fox MD (2005). The human brain is intrinsically organized into dynamic, anticorrelated functional networks. Proc. Natl Acad. Sci. USA.

[3] Damoiseaux JS (2006). Consistent resting-state networks across healthy subjects. Proc. Natl Acad. Sci. USA.

[4] Margulies DS (2016). Situating the default-mode network along a principal gradient of macroscale cortical organization. Proc. Natl Acad. Sci. USA.

[5] Barch DM (2013). Brain network interactions in health and disease. Trends Cogn. Sci..

[6] Northoff G, Duncan NW, Hayes DJ (2010). The brain and its resting state activity–experimental and methodological implications. Prog. Neurobiol..

[7] Dehaene S, Changeux JP (2011). Experimental and theoretical approaches to conscious processing. Neuron.

[8] Spoormaker VI (2010). Development of a large-scale functional brain network during human non-rapid eye movement sleep. J. Neurosci..

[9] Altmann A (2016). Validation of non-REM sleep stage decoding from resting state fMRI using linear support vector machines. NeuroImage.

[10] Schröter MS (2012). Spatiotemporal reconfiguration of large-scale brain functional networks during propofol-induced loss of consciousness. J. Neurosci..

[11] Boveroux P (2010). Breakdown of within- and between-network resting state functional magnetic resonance imaging connectivity during propofol-induced loss of consciousness. Anesthesiology.

[12] Stamatakis EA, Adapa RM, Absalom AR, Menon DK (2010). Changes in resting neural connectivity during propofol sedation. PLoS One.

[13] Stam CJ (2014). Modern network science of neurological disorders. Nat. reviews. Neurosci..

[14] Bullmore E, Sporns O (2009). Complex brain networks: graph theoretical analysis of structural and functional systems. Nat. reviews. Neurosci..

[15] Sporns O, Betzel RF (2016). Modular Brain Networks. Annu. Rev. Psychol..

[16] Vatansever D, Menon DK, Manktelow AE, Sahakian BJ, Stamatakis EA (2015). Default mode dynamics for global functional integration. J. Neurosci..

[17] Gallen CL (2016). Modular Brain Network Organization Predicts Response to Cognitive Training in Older Adults. PLoS One.

[18] Tomasi D, Volkow ND (2011). Association between functional connectivity hubs and brain networks. Cereb. cortex.

[19] van den Heuvel MP, Sporns O (2013). Network hubs in the human brain. Trends Cogn. Sci..

[20] van den Heuvel MP, Sporns O (2011). Rich-club organization of the human connectome. J. Neurosci..

[21] Crossley NA (2013). Cognitive relevance of the community structure of the human brain functional coactivation network. Proc. Natl Acad. Sci. USA.

[22] Grayson DS (2014). Structural and functional rich club organization of the brain in children and adults. PLoS One.

[23] Ball G (2014). Rich-club organization of the newborn human brain. Proc. Natl Acad. Sci. USA.

[24] Achard S (2012). Hubs of brain functional networks are radically reorganized in comatose patients. Proc. Natl Acad. Sci. USA.

[25] Jao T (2015). Functional brain network changes associated with clinical and biochemical measures of the severity of hepatic encephalopathy. NeuroImage.

[26] Schrouff J (2011). Brain functional integration decreases during propofol-induced loss of consciousness. NeuroImage.

[27] Alkire MT (2008). Loss of effective connectivity during general anesthesia. Int. Anesthesiol. Clin..

[28] Tononi G (2008). Consciousness as integrated information: a provisional manifesto. Biol. Bull..

[29] Baars BJ (2002). The conscious access hypothesis: origins and recent evidence. Trends Cogn. Sci..

[30] Brown EN, Purdon PL, Van Dort CJ (2011). General anesthesia and altered states of arousal: a systems neuroscience analysis. Annu. Rev. Neurosci..

[31] Huotari AM (2004). Evoked EEG patterns during burst suppression with propofol. Br. J. Anaesth..

[32] Franks NP (2008). General anaesthesia: from molecular targets to neuronal pathways of sleep and arousal. Nat. reviews. Neurosci..

[33] Monti MM (2013). Dynamic change of global and local information processing in propofol-induced loss and recovery of consciousness. PLoS Comput. Biol..

[34] Barttfeld P (2015). Factoring the brain signatures of anesthesia concentration and level of arousal across individuals. NeuroImage. Clin..

[35] Guldenmund P, Vanhaudenhuyse A, Boly M, Laureys S, Soddu A (2012). A default mode of brain function in altered states of consciousness. Arch. Ital. Biol..

[36] Heine L (2012). Resting state networks and consciousness: alterations of multiple resting state network connectivity in physiological, pharmacological, and pathological consciousness States. Front. Psychol..

[37] Sämann PG (2011). Development of the brain’s default mode network from wakefulness to slow wave sleep. Cereb. cortex.

[38] Martuzzi R, Ramani R, Qiu M, Rajeevan N, Constable RT (2010). Functional connectivity and alterations in baseline brain state in humans. NeuroImage.

[39] Greicius MD (2008). Persistent default-mode network connectivity during light sedation. Hum. Brain Mapp..

[40] Demertzi, A. *et al*. Intrinsic functional connectivity differentiates minimally conscious from unresponsive patients. *Brain* (2015). 10.1093/brain/awv169 26117367

[41] Demertzi A (2014). Multiple fMRI system-level baseline connectivity is disrupted in patients with consciousness alterations. Cortex; a J. devoted study Nerv. Syst. Behav..

[42] Guldenmund P (2013). Thalamus, brainstem and salience network connectivity changes during propofol-induced sedation and unconsciousness. Brain connectivity.

[43] Menon V, Uddin LQ (2010). Saliency, switching, attention and control: a network model of insula function. Brain Structure Funct..

[44] Pappas I, Adapa RM, Menon DK, Stamatakis EA (2018). Brain network disintegration during sedation is mediated by the complexity of sparsely connected regions. NeuroImage.

[45] Shulman GL (1997). Common Blood Flow Changes across Visual Tasks: II. Decreases in Cerebral Cortex. J. Cognit. Neurosci..

[46] Krieger-Redwood K (2016). Down but not out in posterior cingulate cortex: Deactivation yet functional coupling with prefrontal cortex during demanding semantic cognition. NeuroImage.

[47] Spreng RN, Grady CL (2010). Patterns of brain activity supporting autobiographical memory, prospection, and theory of mind, and their relationship to the default mode network. J. Cognit. Neurosci..

[48] Horn A, Ostwald D, Reisert M, Blankenburg F (2014). The structural-functional connectome and the default mode network of the human brain. NeuroImage.

[49] de Pasquale F (2012). A cortical core for dynamic integration of functional networks in the resting human brain. Neuron.

[50] Leech R, Sharp DJ (2014). The role of the posterior cingulate cortex in cognition and disease. Brain.

[51] van den Heuvel MP, Stam CJ, Kahn RS, Hulshoff Pol HE (2009). Efficiency of functional brain networks and intellectual performance. J. Neurosci..

[52] Lambon Ralph, M. A., Jefferies, E., Patterson, K. & Rogers, T. T. The neural and computational bases of semantic cognition. *Nature reviews. Neuroscience* (2016). 10.1038/nrn.2016.150 27881854

[53] Adapa RM, Davis MH, Stamatakis EA, Absalom AR, Menon DK (2014). Neural correlates of successful semantic processing during propofol sedation. Hum. Brain Mapp..

[54] Ashburner J, Friston KJ (2005). Unified segmentation. NeuroImage.

[55] Alakorkko T, Saarimaki H, Glerean E, Saramaki J, Korhonen O (2017). Effects of spatial smoothing on functional brain networks. Eur. J. Neurosci..

[56] Whitfield-Gabrieli S, Nieto-Castanon A (2012). Conn: a functional connectivity toolbox for correlated and anticorrelated brain networks. Brain connectivity.

[57] Behzadi Y, Restom K, Liau J, Liu TT (2007). A component based noise correction method (CompCor) for BOLD and perfusion based fMRI. NeuroImage.

[58] Murphy K, Birn RM, Handwerker DA, Jones TB, Bandettini PA (2009). The impact of global signal regression on resting state correlations: are anti-correlated networks introduced?. NeuroImage.

[59] Fair DA (2007). A method for using blocked and event-related fMRI data to study “resting state” functional connectivity. NeuroImage.

[60] Power JD, Barnes KA, Snyder AZ, Schlaggar BL, Petersen SE (2012). Spurious but systematic correlations in functional connectivity MRI networks arise from subject motion. Neuro Image.

[61] Power JD (2011). Functional network organization of the human brain. Neuron.

[62] Uddin LQ, Kelly AM, Biswal BB, Castellanos FX, Milham MP (2009). Functional connectivity of default mode network components: correlation, anticorrelation, and causality. Hum. Brain Mapp..

[63] Rubinov M, Sporns O (2011). Weight-conserving characterization of complex functional brain networks. NeuroImage.

[64] Rubinov M, Sporns O (2010). Complex network measures of brain connectivity: uses and interpretations. NeuroImage.

[65] Blondel VD, Guillaume J-L, Lambiotte R, Lefebvre E (2008). Fast unfolding of communities in large networks. J. Stat. Mech-Theory E.

[66] Guimera R, Amaral LA (2005). Cartography of complex networks: modules and universal roles. J. Stat. Mech..

[67] Zhou S, Mondragon RJ (2004). The rich-club phenomenon in the Internet topology. IEEE Commun. Lett..

[68] Betzel RF, Bassett DS (2018). Specificity and robustness of long-distance connections in weighted, interareal connectomes. Proc. Natl Acad. Sci. USA.

[69] Betzel RF (2017). The modular organization of human anatomical brain networks: Accounting for the cost of wiring. Network. Neurosci..

[70] Xia M, Wang J, He Y (2013). BrainNet Viewer: a network visualization tool for human brain connectomics. PLoS One.

